# Immediate and six-week effects of wearing a knee sleeve following anterior cruciate ligament reconstruction on knee kinematics and kinetics: a cross-over laboratory and randomised clinical trial

**DOI:** 10.1186/s12891-022-05488-2

**Published:** 2022-06-10

**Authors:** Gisela Sole, Peter Lamb, Todd Pataky, Anupa Pathak, Stefan Klima, Pierre Navarre, Niels Hammer

**Affiliations:** 1 Centre for Health, Activity and Rehabilitation Research, School of Physiotherapy, University of Otago, Dunedin, New Zealand; 2 School of Physical Education, Sport and Exercise Sciences, University of Otago, Dunedin, New Zealand; 3 Graduate School of Medicine, Department of Human Health Sciences, Kyoto University, Kyoto, Japan; 4 School of Public Health, Faculty of Medicine and Health, University of Sydney, New South Wales, Australia; 5 Orthopaedicus, Leipzig, Germany; 6 Southland Hospital, Invercargill, New Zealand and Clinical Senior Lecturer, University of Otago, Dunedin, New Zealand; 7 Division of Macroscopic and Clinical Anatomy, Gottfried Schatz Research Center, Medical University of Graz, Graz, Austria; 8 Department of Orthopaedic and Trauma Surgery, University of Leipzig, Leipzig, Germany; 9 Fraunhofer IWU, Medical Branch, Dresden, Germany

**Keywords:** Anterior cruciate ligament reconstruction, Hopping, Kinematics, Kinetics, Flexion angle trajectory, Knee sleeve

## Abstract

**Background:**

Elastic knee sleeves are often worn following anterior cruciate ligament reconstruction (ACLR) but their effects on movement patterns are unclear.

**Aim:**

To determine the immediate and six-week effects of wearing a knee sleeve on biomechanics of the knee during a step-down hop task.

**Methods:**

Using a cross-over design, we estimated sagittal plane knee kinematics and kinetics and stance duration during a step-down hop for 31 participants (age 26.0 [SD 6.6] years, 15 women) after ACLR (median 16 months post-surgery) with and without wearing a knee sleeve. In a subsequent randomised clinical trial, participants in the ‘Sleeve Group’ (*n* = 9) then wore the sleeve for 6 weeks at least 1 h daily, while a ‘Control Group’ (*n* = 9) did not wear the sleeve. We used statistical parametric mapping to compare (1) knee flexion/extension angle and external flexion/extension moment trajectories between three conditions at baseline (uninjured side, unsleeved injured side and sleeved injured side); (2) within-participant changes for knee flexion angles and external flexion/extension moment trajectories from baseline to follow-up between groups. We compared discrete flexion angles and moments, and stance duration between conditions and between groups.

**Results:**

Without sleeves, knee flexion was lower for the injured than the uninjured sides during mid-stance phase. When wearing the sleeve on the injured side, knee flexion increased during the loading phase of the stance phase. Discrete initial and peak knee flexion angles increased by (mean difference, 95% CIs) 2.7° (1.3, 4.1) and 3.0° (1.2, 4.9), respectively, when wearing the knee sleeve. Knee external flexion moments for the unsleeved injured sides were lower than the uninjured sides for 80% of stance phase, with no change when sleeved. The groups differenced for within-group changes in knee flexion trajectories at follow-up. Knee flexion angles increased for the Control group only. Stance duration decreased by 22% for the Sleeve group from baseline to follow-up (-89 ms; -153, -24) but not for the Controls.

**Conclusions:**

Application of knee sleeves following ACLR is associated with improved knee flexion angles during hop landing training. Longer term (daily) knee sleeve application may help improve hop stance duration, potentially indicating improved hop performance.

**Trial registration:**

The trial was prospectively registered with the Australia New Zealand Clinical Trials Registry No: ACTRN12618001083280, 28/06/2018. ANZCTR

**Supplementary Information:**

The online version contains supplementary material available at 10.1186/s12891-022-05488-2.

## Introduction

Rehabilitation following anterior cruciate ligament (ACL) reconstruction includes progressive exercise prescription to improve range of motion, muscle strength, sensorimotor control and sports- and work-specific skills, as well as physical fitness [1]. Psychosocial factors, such as fear of re-injury, are also considered and strategies are included to improve confidence and self-efficacy for return to physical activity [2, 3]. Such strategies may include use of a knee sleeve [2, 4, 5]. Mechanisms underlying potential effects of use of such knee sleeves are unclear.

Residual changes or side-to-side asymmetries in movement-related biomechanics have been reported following ACL reconstruction. Such differences include less knee flexion and lower knee joint moments during walking, stair ascent and descent [6, 7], and jumping tasks [8, 9]. Jump-landing strategies have received substantial attention as a risk factor for ACL rupture and as outcomes following such injury [10, 11]. In general, landing with less hip, knee and ankle flexion, thus a more ‘extended knee’, indicates a ‘stiffer’ leg with higher stress on the ACL [9], potentially increasing risk of re-injury or sub-optimal recovery following injury [12].

Various interventions have been explored to improve lower limb biomechanics during jump-landing, including plyometric training with or without verbal and augmented feedback [11, 13, 14]. Jump landing training can lead to immediate increased knee flexion and vertical ground reaction forces during jump landing, with less influence on knee external flexion moments [11]. It is still unclear to what extent such training influences movement patterns over the longer term [15].

Elastic or neoprene sleeves may be used during rehabilitation post-ACL reconstruction [5]. Such sleeves do not limit range of motion but are thought to influence sensorimotor control [16], potentially leading to enhanced movement patterns or knee joint position sense [16–18]. Wearing knee sleeves may improve the individual’s knee-related confidence [19–21]. Results of laboratory studies suggest that using such sleeves may improve gait- and function-related performance for people with symptomatic knee osteoarthritis [18], knee-healthy participants [20, 22] and elite athletes [23]. Knee sleeves may be at the cost of reduced knee flexion during drop landing, as shown in laboratory-based study of immediate effects with knee-healthy participants [22]. Kuster et al. [21] reported that wearing an elastic sleeve led to increased vertical ground reaction (GRF) during a drop jump for participants with ACL reconstructions, as well as improved balance, evident with enhanced steadiness during the one-legged stance. Little is known about the potential influences of knee sleeves on movement patterns at the knee for participants with ACL reconstruction, particularly knee flexion and knee joint moments, across an extended period of use.

We previously reported that wearing a knee sleeve, on average, led to an immediate improved distance of a single leg hop in participants with residual self-reported functional limitations following ACL reconstruction [24]. However, we found no significant differences over a six-week period between a smaller group of participants wearing the sleeve at least one hour daily compared to a control group. Similarly, no between-group differences were found for self-reported knee function (assessed with the International Knee Disability Committee – Short Knee Form, IKDC-SKF), and thigh muscle strength at follow-up [24].

The aim of this second study was to determine immediate and six-week effects of wearing a knee sleeve on knee kinematic, kinetic and temporal variables in participants with an ACL reconstruction during a standardised step-down hop. The primary research hypothesis (H1) was that knee flexion and knee external flexion moments would increase during the stance phase of the task when wearing a sleeve compared to not wearing the sleeve. Secondary hypotheses were that the group wearing the sleeve for a six-week period would have increased knee flexion angles and knee flexion moments during the stance phase than the control group (H2), and that stance duration of the step-down-hop would decrease while wearing the sleeve compared to not wearing the sleeve, suggesting improved coordination and performance of the submaximal countermovement hop (H3).

## Methods

Data were collected during two sessions (baseline and six-week follow-up) in a university research laboratory and via REDCap (Research Electronic Data Capture, hosted by the University of Otago, Dunedin, New Zealand). Ethical approval was granted by the Health and Disability Ethics Committee (of New Zealand). We followed CONSORT reporting guidelines [25]. The sample of this study was the same sample as in the previous report [24].

### Trial design and binding

Part 1 of the study consisted of a cross-over laboratory-based study, exploring immediate effects of wearing the knee sleeve. It was impossible to blind participants and assessors to the sleeve condition in Part 1. Part 2 was a parallel two-armed, assessor-blinded randomised clinical trial (RCT), with the same participants as in Part 1 [24]. The biostatistician and the research assistant were blinded to group allocation for the RCT.

### Participants

#### Recruitment

We recruited participants via community advertising and using TrialFacts (https://trialfacts.com/) from September 2018 to September 2020, the end of the funding period. Volunteers completed a questionnaire (also serving as screening for eligibility) via REDCap prior to attending the laboratory session [5]. The questionnaire included demographics, injury and surgery history, the International Knee Documentation Committee Subjective Knee Form (IKDC-SKF) [26] and the Tegner activity scale [27].

#### Inclusion criteria

We recruited men and women, aged 18–40 years, who underwent ACL reconstruction within 6 months to 5 years previously [5]. We targeted individuals who had not yet reached full functional level, for the purpose of this study defined by a score between 40 to 80/100 on the IKDC-SKF [26, 28, 29]. Due to slow recruitment rate, and amendment was approved by the ethics committee to expand the duration since the ACL reconstruction from 3 to 5 years.

#### Exclusion criteria

We excluded participants who had undergone a revision ACL reconstruction of the same knee (due to re-injury), or a previous ACL reconstruction of the opposite knee; self-reported any other lower limb, pelvic or low back musculoskeletal injuries or disorders that required medical care over the past 6 months; had known systemic, neurological or cardiovascular disorders; or had a body mass index (BMI) greater than 30 kg/m^2^ [5]. We excluded participants with an IKDC-SKF score less than 40 (due to potential safety risk during the laboratory-based tasks) or greater than 80/100 (as use of a sleeve would clinically be less likely to add benefit) [5].

### Procedures

#### Randomisation

We randomised each participant twice (once for the cross-over trial, and once for the RCT) with equal numbers in each group for both allocations. The research officer sequentially block randomised groups of 8 participants with an electronic random number generator before participants entered the study. Each group was stratified by sex. The research officer informed the assessor for the laboratory data collection of the order for the conditions for the cross-over trial, and the group allocation (for the RCT) via email prior to the participant’s first laboratory session [5]. Participants provided written informed consent at the start of the first session. Participants were dressed in a singlet, a pair of shorts and their own sport shoes. Body mass and height were measured during the baseline session [5].

#### Part 1: Laboratory cross-over trial

Participants undertook two hopping tasks: a maximum horizontal single leg hop and a sub-maximum step-down hop. A sub-maximal level was chosen for safety as we were seeking participants with remaining residual functional limitations (as defined by the IKDC-SKF). To allow comparison of knee mechanics across the six-week period, the distance of the required hop was standardised and individualised based on the participants single-leg hop distance of the uninjured side.

Participants practised the hopping tasks at sub-maximal distance with the uninjured and injured sides until they were confident with performing them as part of familiarisation and warm-up. They performed the maximum horizontal hop prior to undertaking the step-down-hop.

#### Part 2 Randomised clinical trial

On completion of the first laboratory session, the assessor informed participants of their group allocation for the RCT. All participants were asked to return to the laboratory following the six-week period to repeat the above assessments, repeating the hopping tasks without wearing the knee sleeve.

### Intervention

We used a commercially available knee sleeve, GenuTrain (Bauerfeind® AG, Zeulenroda-Triebes, Germany), a CE-certified medical device, as the intervention. The sleeve consists of flexible elastic/knitted materials with an in-built gel pad around the patella margins. It was designed to provide knee support, potentially enhancing knee proprioception, without limiting range of motion. All participants performed the step-down hop with and without the sleeve for the cross-over trial (Part 1). For Part 2 (RCT), only participants of the ‘Sleeve Group’ (intervention) were provided with the knee sleeve for the six-week period. They were instructed to wear the sleeve for a minimum of 1 h per day during their rehabilitative exercises, physical activity and sports. The control group were not provided with a sleeve during this period. As reported previously [24], participants of the ‘Sleeve Group’ were asked to document the use of the knee sleeve in a daily diary (Microsoft® Excel spreadsheet) and all participants were asked to document their daily physical activity and exercise.

The researcher explained the use of the knee sleeve to the ‘Sleeve Group’ and provided them with an instructional leaflet. They were informed to discontinue use of the knee sleeve and contact the researcher should any side-effects evolve, such as discomfort during use, pain, burning sensations of the knee, leg or foot, are swelling of the knee or calf [5].

### Outcomes

**Fig. 1:**
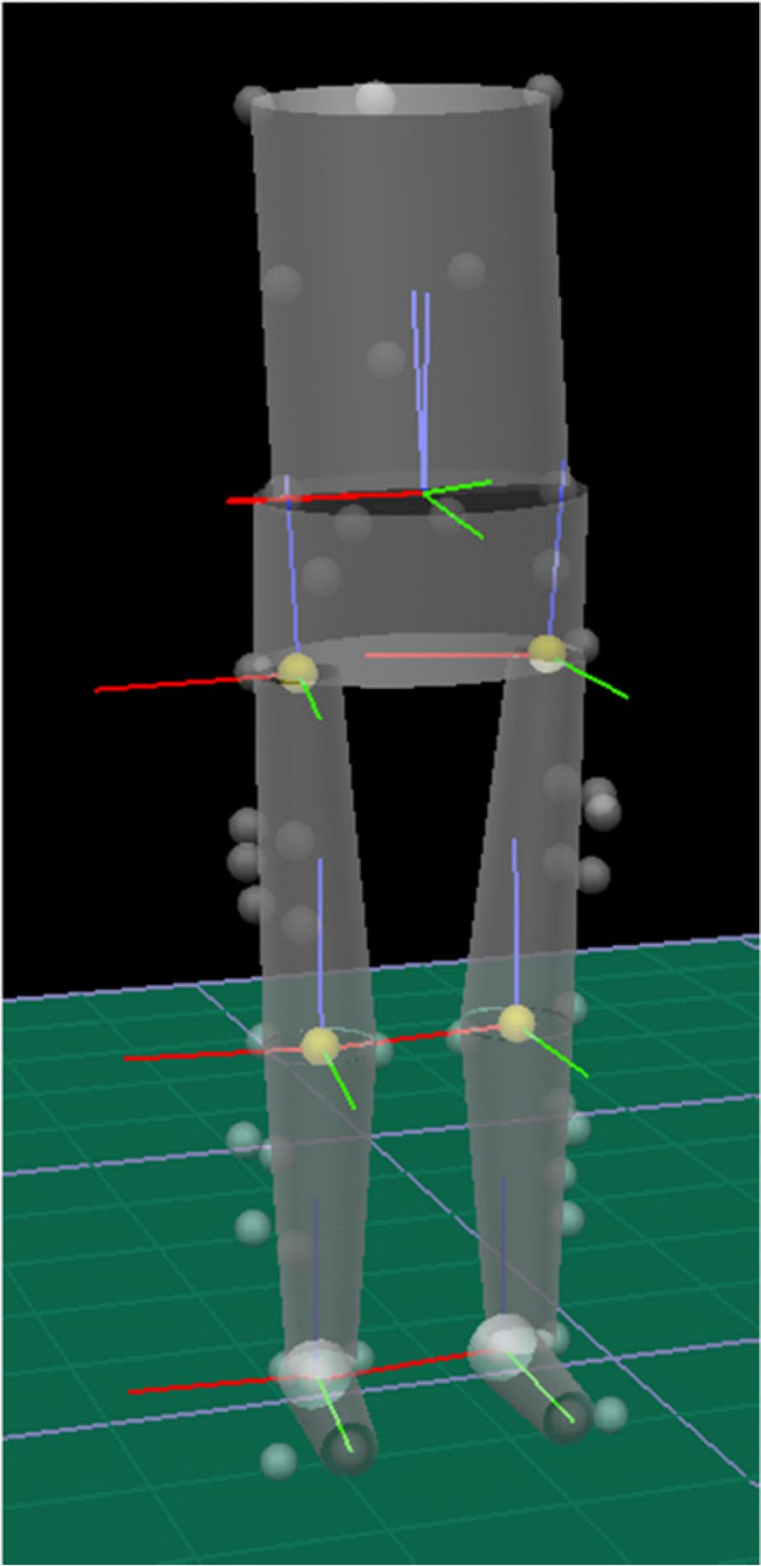
Placement of reflective markers: acromia, C7, T10, inferior scapular angles, posterior iliac spines, iliac crests, greater trochanter, medial and lateral knee, medial and lateral malleoli, a 5-marker cluster on the later thigh, a 4-marker cluster on the lateral shin, heel of shoe, metatarsal head 1, base of metatarsal 5

- 1) Single-leg horizontal jump: After familiarisation with the tasks, they performed 3 trials of single-leg maximum horizontal jump, as described in the first report [24]. The maximum jump distance of the uninjured side during Session 1 was used to calculate the individualised target for the hop distance during the step-down task. The target step-down hop distance was 60–70% of the maximum single leg hop distance of the uninjured side, and was kept constant for the participant across the two sessions.
- 2) Step-down hop: Three-dimensional motion analysis was performed for the step-down hop with 11 infra-red cameras (Motion Analysis Corporation, Santa Rosa, CA, USA), sampling at 120 Hz, and Cortex 2.0 software, synchronized with a floor-mounted force plates (BP2436 AMTI Inc., Newton, MA, USA), sampling at 2,400 Hz. A set of 42 reflective markers (diameter 12.5 mm) were applied to the trunk, pelvis and lower extremities (Fig. 1). The marker positions were tracked by the camera system, reconstructed in 3D space and used to define segment coordinate systems.

Following the marker placement, a static anatomical calibration trial was performed with the participants standing still, on both feet. A functional movement trial was then performed by moving the hip in the three planes for calculation of functional hip joint centres [30]. One trial was undertaken for each side respectively.

**Fig. 2:**
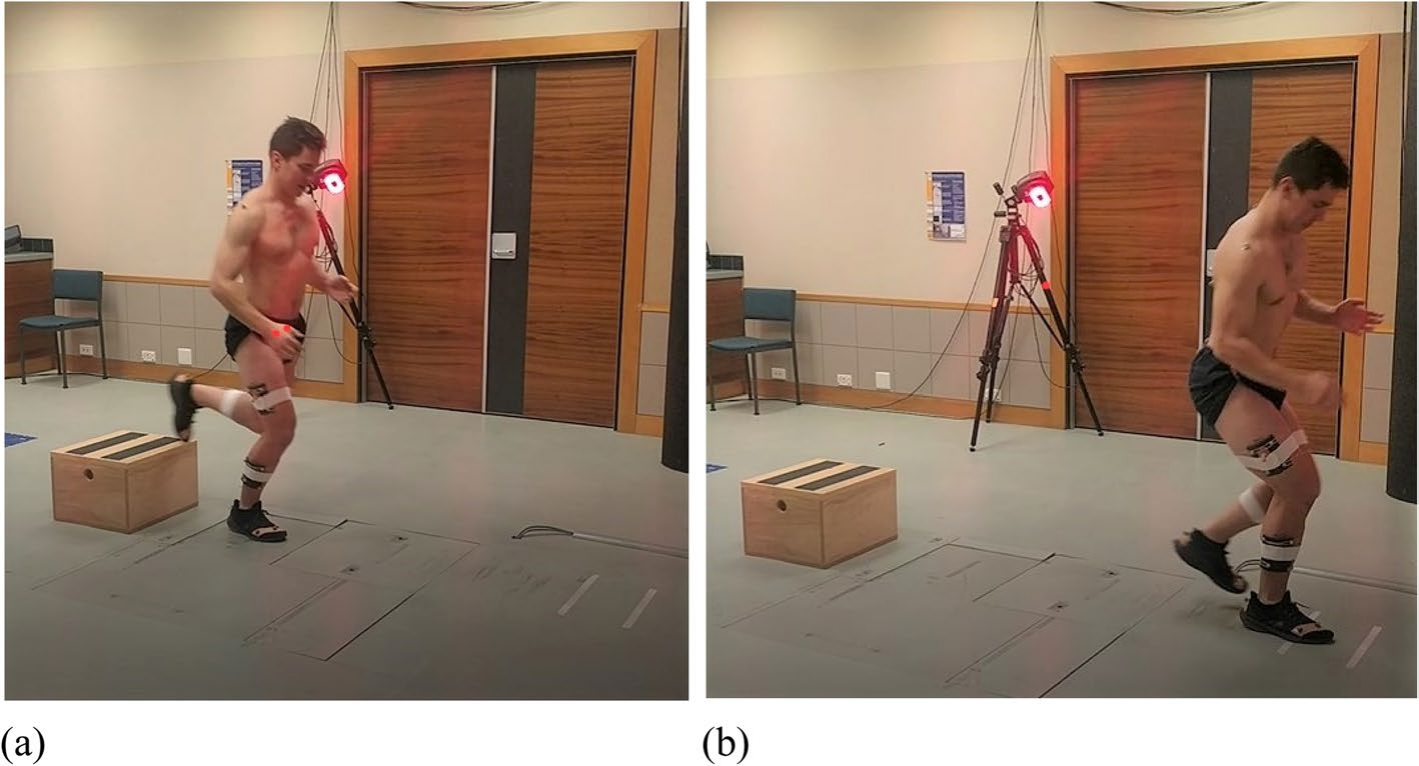
The participant is performing the step-down hop with the right side. **a** Stance phase on the floor-embedded force plate; (**b**) hop landing at 60–70% of maximum horizontal jump distance, marked by two strips on the floor

The participants were then asked to stand on a 30-cm box, placed 15 cm from the force plate, and performed a step-down hop (adapted from E Kristianslund and T Krosshaug [31]) onto the force plate: the participant was asked to step off the box with either the injured or the uninjured leg onto the force plate (Fig. 2A), then hop forward off the plate as fast as possible. The distance of that hop was 60–70% of the maximum horizontal jump distance of the uninjured side (Fig. 2B). They performed the step-down-hop with the uninjured side first, then the injured side under the (1) ‘control’ condition (no sleeve) and (2) the ‘sleeve’ condition (experimental, wearing the sleeve), ordered by randomisation. A 5-min walk between the conditions provided a standardised run-in to the second condition to minimise carryover effects.

The outcome variables during the stance phase of the hop were knee flexion angles at initial contact, peak knee flexion and external flexion moments, and stance duration. We found intraclass correlations coefficients (ICCs) above 0.9 for knee kinematics and kinetics, and 0.89 for stance duration (for 10 knee-health participants). The standard error of measurements (SEM) and smallest detectable differences (SDD) are provided in Additional file 1: Appendix 1.

### Data processing and analysis

Cortex 5.5 (Motion Analysis Corporation, Santa Rosa, CA, USA) was used to track and label the markers, and the biomechanical model, kinematic (joint angles) and kinetic (moments) data were calculated using Visual3D Professional v6 (C-Motion, Inc., Germantown, MD, USA). Functional hip joint centres were estimated based on a movement trial involving all degrees of freedom of the hip [32], while anatomical joint centres at the knee and ankle joints were based on medial and lateral markers [30]. Marker clusters attached to a rigid base were fixed to the thighs and shanks to minimise marker coordinate estimation error [33]. Kinematic and kinetic data were filtered using a low-pass, double, second order Butterworth filter with a cut-off of 10 Hz.

Stance duration was the time from start to end of stance phase, defined by the vertical component of the ground reaction force exceeding and returning below 20 N, respectively. Peak knee flexion angles and flexion angles at initial contact were extracted for the stance phase. Based on the biomechanical model’s segment inertial properties and joint properties, inverse dynamics analyses were applied to the model kinematics and ground reaction forces to calculate the net joint moments. Net joint moments were resolved into the proximal segment’s coordinate system and represented as vectors. The moments were normalised to body size (Nm/BW*HT). For all variables, the averages of five trials for each limb (injured versus uninjured) and condition (sleeved and unsleeved) for each participant were calculated.

### Sample size

We report a secondary analysis of a larger study investigating effects of wearing a knee sleeve for individuals following ACL reconstruction. The sample size was based on the primary outcome measure, the horizontal hop distance [24]. We did not undertake a sample size calculation specifically for the outcomes reported in the current paper.

### Data analysis

Demographic data were presented descriptively (means and standard deviations for approximately normally distributed continuous variables; medians and ranges for other continuous variables; and counts and percentages for categorical variables).

#### Statistical parametric mapping

We analysed kinetic and kinematic trajectories using Statistical Parametric Mapping (SPM, http://spm1d.org/; Pataky, 2012) [34]. SPM allows comprehensive statistical analyses of the trajectory of a given biomechanical variable (such as knee angles) during a specific task, using the entire *n*-dimensional biomechanical sampling data. That contrasts with discrete point analyses, such as peak knee flexion, that assess only the one point during the entire movement. SPM uses Random Field Theory to make probabilistic conclusions based on the random behaviour of that 1D observational unit. Scalar observations of discrete variables are based on traditional 0D Gaussian randomness [35]. Thus, the whole trajectory can be assessed with SPM, and hypotheses relating to differences at specific time points during the task (such as a hop) do not need to be defined a priori.

Stance phase kinematic and kinetic trajectories in the sagittal plane were temporally normalised to stance duration using linear interpolation across 100 equally spaced time points. Trajectories of five trials for each limb and each session were computed using MATLAB R2018b (The MathWorks Inc., Natick, MA, USA). The mean trajectory for the uninjured and injured sides (sleeved and unsleeved conditions, Session 1) were then computed.

For Part I, one-way repeated measures ANOVA was used to compare three conditions at the baseline: (a) uninjured side, unsleeved to (b) injured side, unsleeved, and (c) injured side, sleeved. ANOVA was conducted separately for each of two dependent variables: (i) knee flexion, and (ii) external flexion moment, and a conservative Bonferroni threshold of 0.025 was adopted to correct for multiple comparisons across these two tests. Post-hoc analyses using paired t tests and a secondary Bonferroni correction across the three pairwise tests (a + b, a + c and b + c) were used to assess between-condition effects.

For Part 2, within-participant changes were depicted between baseline and follow-up sessions (unsleeved only). An SPM independent t test was used to compare within-participant changes in the Sleeve group versus the Control Group. This t test was repeated for each of the two aforementioned dependent variables, again with a conservative Bonferroni correction of 0.025.

#### Discrete variable analysis

Post hoc analyses were also performed for the discrete variables. Analyses for Part 1 (cross-over trial) were adjusted for participant sex, surgery type, and time since surgery (as a continuous measure), and sequence effect. We used linear mixed models using Restricted Maximum Likelihood (REML) to estimate random effects for the analyses for the knee flexion angles (angle at initial contact, peak angle, and excursion), knee peak external flexion moment and stance duration. A random measurement occasion effect (both limbs measured at multiple time points) and a random participant effect were nested within participant. We performed those analyses with Stata (*16.1*, *StataCorp* LLC, College Station, TX, USA).

For Part 2 (RCT), individual change scores from baseline to follow-up were calculated for knee flexion angles, moments and stance duration. The change scores were compared between the Sleeve Group and the Control Group using Mann–Whitney U tests for each outcome. The alpha level was set at *p* ≤ 0.05. These analyses and those of demographic data were performed with SPSS Version 24.0 (IBM Corp, Armonk, NY).

## Results

**Fig. 3:**
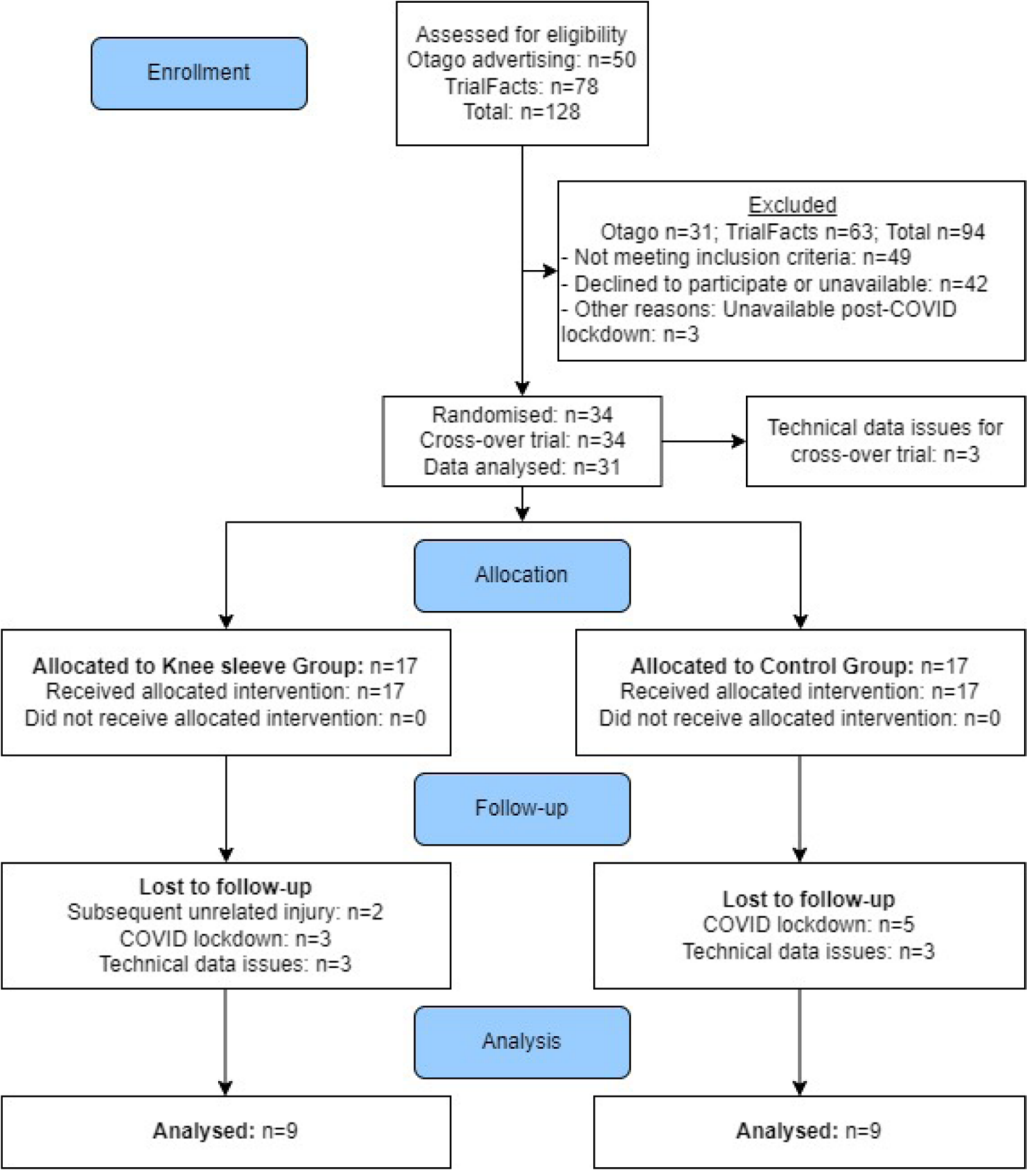
Flowchart of participant recruitment, allocation and follow-up. * Participants were lost to the laboratory-based follow-up data collection due to the COVID-19 lockdown in March/April 2020. IKDC-SKF: International Knee Documentation Committee Subjective Knee Form

We assessed 34 participants at baseline, but data for three participants were excluded due to technical issues. Two participants of the Sleeve Group withdrew from the study following baseline assessment due to knee re-injuries, unrelated to use of the knee sleeve (Fig. 3). Eight participants were lost to follow-up due to the COVID-19 lockdown in New Zealand, March/April 2020. Twenty-four participants completed the follow-up laboratory session. Data from six participants was excluded due to technical difficulties, resulting in data being analysed for nine participants in each group for Part 1 (RCT). Demographic data of the participants are provided in Table 1.

**Table 1:** Demographic data (*n* = 31)

	All	Men	Women
Men/Women n (%)	31	16 (52)	15 (48)
Age (years)	26.0 (6.6)	24.6 (5.6)	27.5 (7.5)
Mass (kg)	76.4 (11.6)	76.9 (11.8)	75.9 (11.8)
Height (m)	1.72 (0.1)	1.76 (0.07)	1.68 (0.08)
Body mass index (kg.m^−2^)	25.8 (3.1)	24.8 (2.6)	26.8 (3.3)
Reconstruction: Hamstring/patella tendon grafts n (%)	15 (48)/16 (52)	9 (56)/7 (44)	6 (35)/9 (64)
Meniscal repair: no/yes n (%)	23 (74)/8 (26)	13 (81)/3 (19)	10 (67)/5 (33)
Time since ACL injury (months)	21 (9 – 108)	21 (9 – 55)	21 (12 – 108)
Time since surgery (months)	16 (6 – 53)	17 (6 – 44)	16 (7 – 53)
Time from ACL injury to surgery (months)	6 (1 – 89)	6 (1 – 11)	8 (1 – 89)
Tegner activity scale: Preinjury (median, range)	8 (3 – 10)	9 (3 – 10)	7 (6 – 10)
Tegner activity scale: Baseline (median, range)	5 (2 – 9)	5 (2 – 9)	4 (2 – 9)
IKDC-SKF Baseline	64.1 (9.6)	69.2 (10.2)	66.1 (9.0)
IKDC-SKF Follow-up	73.7 (11.5)	71.3 (13.2)	76.5 (8.9)

Figures are numbers (Frequency), Mean (SD) or Medians (minimum – maximum)

*IKDC-SKF* International Knee Documentation Committee Subjective Knee Form

Due to the large rate of participants lost to follow-up for the biomechanical data (42% of 31 participants), we compared the IKDC-SKF at baseline and at follow-up between those whose data were included in the results to those that were excluded or did not attend the second laboratory session. There was no statistically significant difference for IKDC-SKF between participants whose data were included in the RCT (*n* = 18; 68.1 [9.3]) and those who were lost to follow-up for the biomechanical data (*n* = 13; 67.1 [10.4], *p* = 0.775). Similarly, there were no differences for the IKDC-SKF at follow-up (included: 73.3 [13.1]; lost to follow-up: 74.2 [8.9], *p* = 0.863).

### Part 1: Immediate effects

**Fig. 4:**
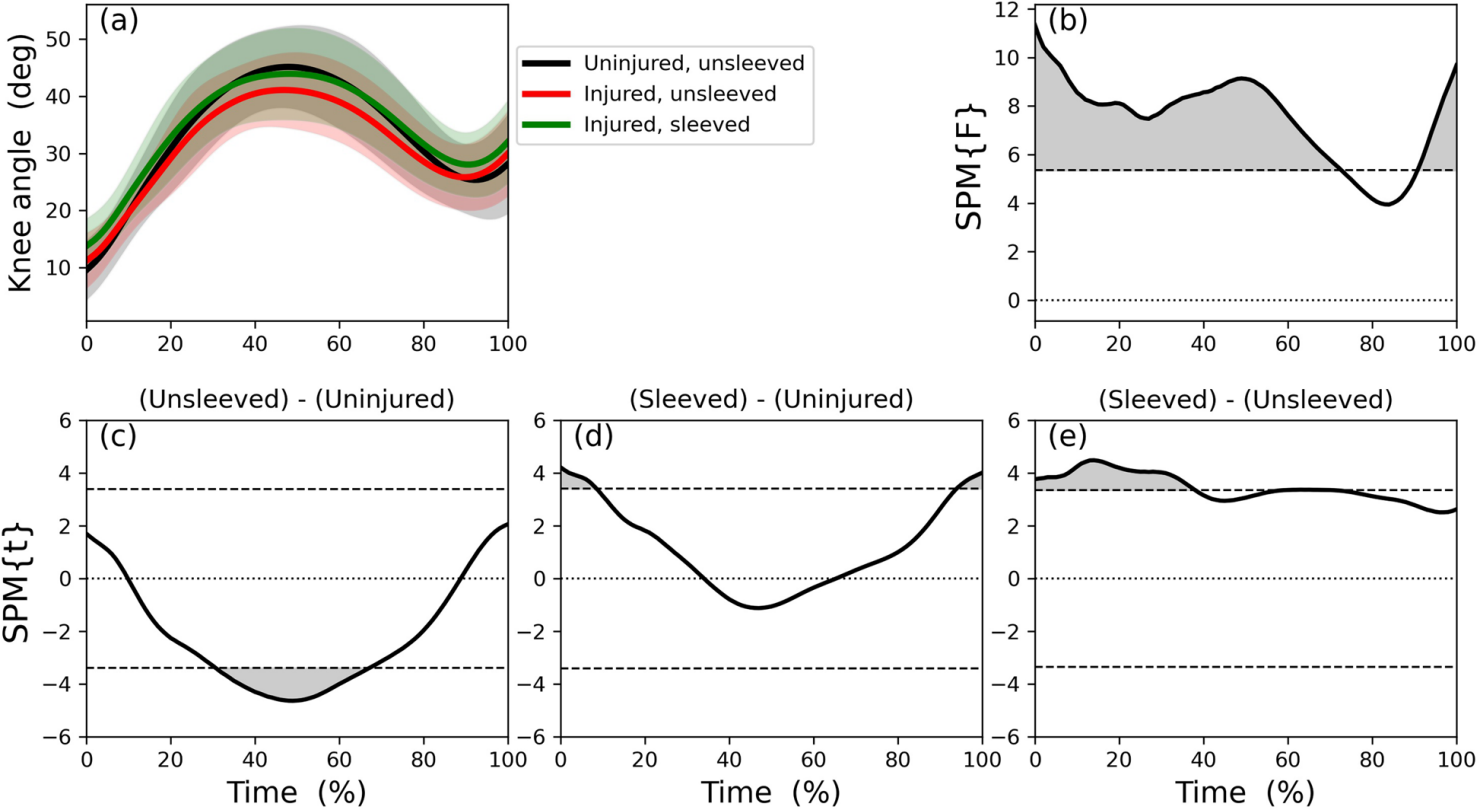
Statistical Parametric Mapping of knee flexion angle (degrees) trajectory during the stance phase of the step-down-and-hop task: (**a**): mean and individual participants’ time series for flexion angle trajectories for uninjured sides, and unsleeved and sleeved injured sides; (**b**): ANOVA on the knee angle trajectory; post-hoc analyses: (**c**): uninjured side versus unsleeved injured; (**d**): uninjured versus sleeved injured; (**e**): injured side, sleeved versus unsleeved conditions

The knee flexion angle trajectories of the unsleeved uninjured side, unsleeved and sleeved injured sides (Fig. 4a) differed across most of the stance phase (Fig. 4b). Those differences were explained mainly by differences during mid-phase stance (during which peak flexion occurs) when comparing the injured to the uninjured sides (Fig. 4c) and during the first third of the stance phase when comparing the injured side sleeved to the unsleeved conditions (Fig. 4e). Those differences were confirmed with post-hoc discrete variable analysis (Table 2): the (unsleeved) injured side had less peak flexion compared to the (unsleeved) uninjured side. When wearing the sleeve, the injured side flexion angle at initial contact and at its peak increased compared to not wearing the sleeve. On average, the total flexion excursion range during stance was less for the injured side compared to the uninjured side, and did not change when wearing a sleeve.

**Table 2:** Immediate effects of wearing the sleeve: cross-over trial (*n* = 31)

	**Unsleeved Condition**		**Sleeved Condition**	**Between side comparison (unsleeved)**		**Between condition comparison, injured side**	
	**Uninjured side**	**Injured side**	**Injured side**	**Mean Difference (95%CI)**	***p*-value**	**Mean Difference (95%CI)**	***p*-value**
Knee flexion angle at initial contact (°)	9.5 (5.3)	11.0 (4.9)	13.5 (4.9)	1.4 (-0.5, 3.3)	0.148	2.7 (1.3, 4.1)	< 0.001
Knee peak flexion angle (°)	46.2 (6.4)	42.5 (5.7)	45.4 (7.1)	-3.9 (-5.7, -2.0)	< 0.001	3.0 (1.2, 4.9)	0.001
Knee flexion/ extension excursion (°)	36.7 (5.1)	31.4 (4.4)	31.9 (6.7)	-5.3 (-7.6, -2.9)	< 0.001	0.4 (-1.3, 2.0)	0.673
Knee peak external flexion moment (Nm/BW.ht)	0.114 (0.029)	0.087 (0.028)	0.093 (0.030)	-0.027 (-0.037, -0.017)	< 0.001	0.007 (0.002, 0.011)	0.005
Stance duration (ms)	380 (79)	376 (68)	395 (85)	-4 (-31, 22)	0.751	19 (-5, 43)	0.127

**Fig. 5:**
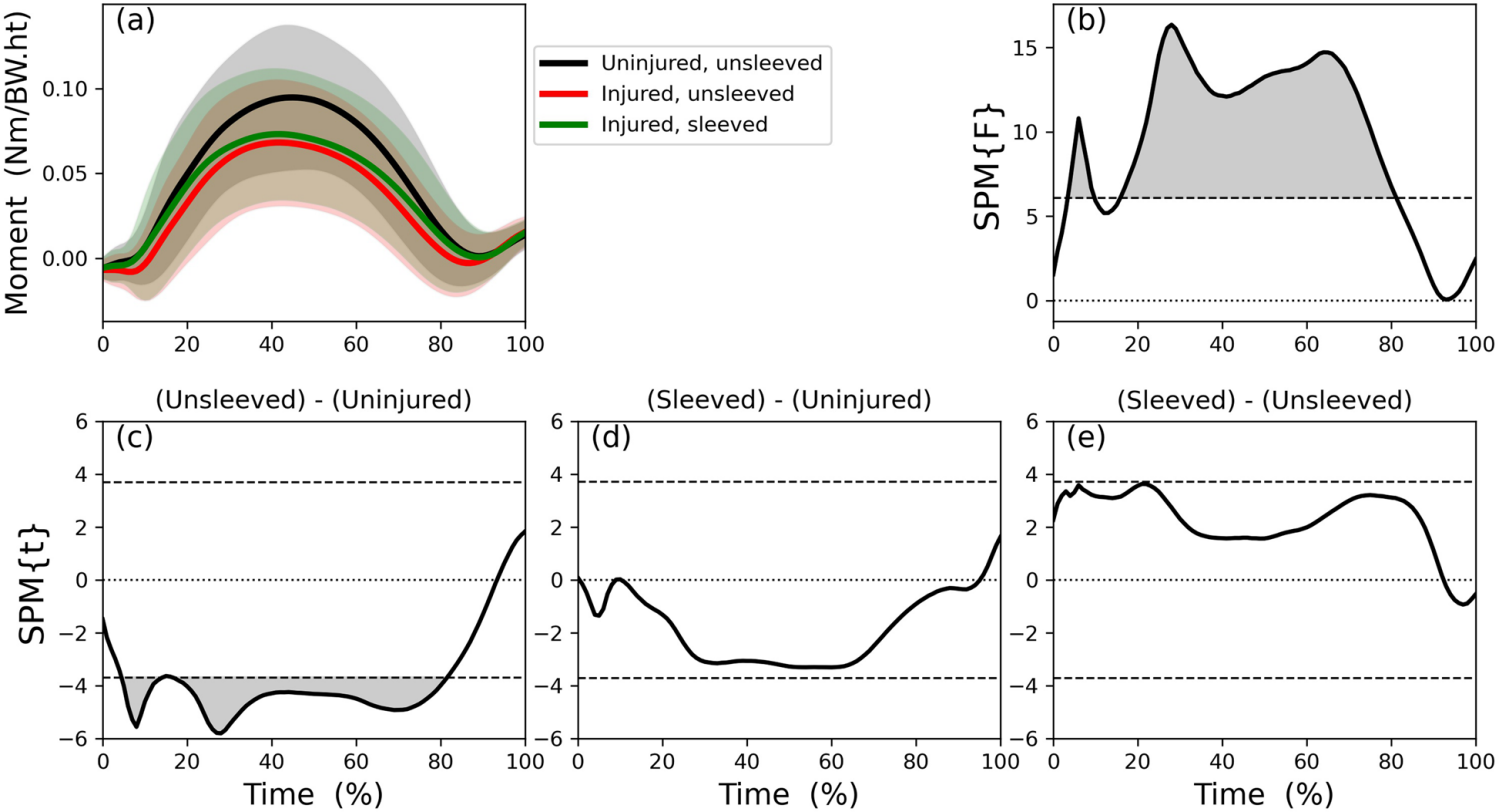
Statistical Parametric Mapping of external knee flexion moment (Nm/BW.ht) trajectory during the stance phase of the step-down-and-hop task: (**a**): mean and individual participants’ time series for external knee flexion moment trajectories for uninjured sides, and unsleeved and sleeved injured sides; (**b**): ANOVA on the knee flexion moment trajectory; Post-hoc analyses: (**c**): uninjured side versus unsleeved injured; (**d**): uninjured versus sleeved injured; (**e**): injured side, sleeved versus unsleeved conditions

The knee external flexion moment trajectory of the unsleeved uninjured side, the unsleeved injured sides and sleeved injured sides (Fig. 5a) also differed across most of the stance phase (Fig. 5b). Those differences were explained by differences between the (unsleeved) uninjured and (unsleeved) injured sides from 5 to 80% of stance (Fig. 5c). The post-hoc discrete variable analysis confirmed that the (unsleeved) injured knee had lower peak knee flexion moments compared to the uninjured side. However, discrete variable analysis contrasted to SPM when comparing injured side peak knee flexion moments while wearing the sleeve to not wearing the sleeve (Fig. 5e; Table 2). Wearing the sleeve, on average, increased the peak knee flexion moments compared to the unsleeved condition (Table 2).

#### Temporal variable

On average, stance duration did not differ between injured and uninjured sides, nor when comparing the sleeved to the unsleeved conditions for the injured sides (Table 2).

### Part 2: Randomised clinical trial

**Fig. 6:**
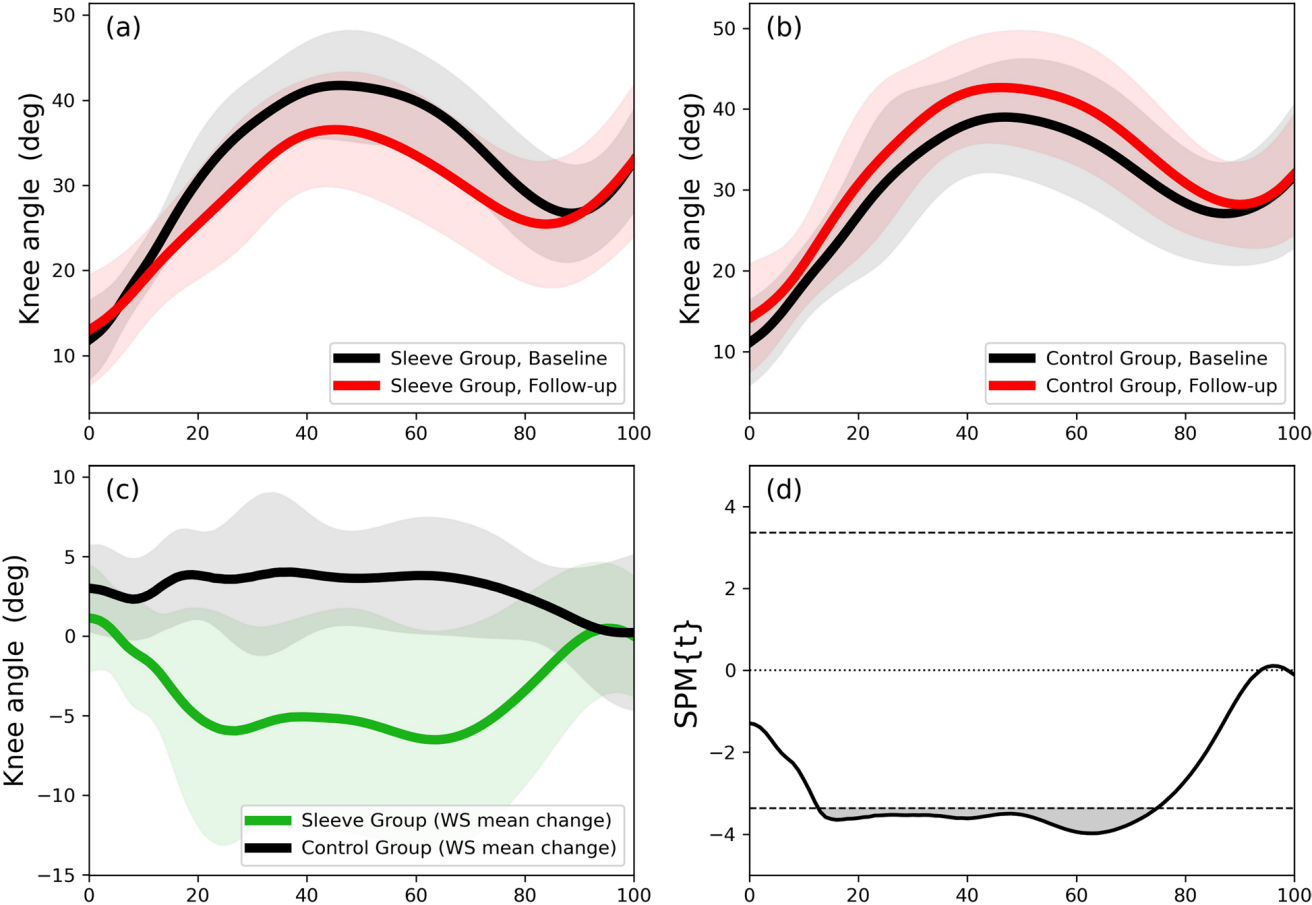
Injured side knee flexion angle trajectory (degrees) during stance of the step-down-hop task for the (**a**) Sleeve Group and (**b**) Control Groups; (**c**) within-participant mean differences between baseline and follow-up for the Sleeve and the Control Groups; (**d**) between-group SPM analysis (independent t tests) of the baseline to follow-up within-participant mean changes. (WS: within-subject)

Data of nine participants in each group could be included in the analysis (Sleeve Group: 5 women, 4 men; Control Group: 3 women, 6 men). Knee angle trajectories for the Sleeve Group and the Control Groups are shown in Fig. 6 at (a) baseline and (b) six-week follow-up. The SPM analysis of the baseline to follow-up mean changes between groups showed significant differences from 10 to 75% of stance phase (Fig. 6d). The Sleeve Group knee trajectory showed larger changes than for the Control Group, leading to less knee flexion at follow-up. The difference is not reflected with discrete variables for flexion (initial contact, peak flexion and excursion, Table 3). However, the 95% confidence intervals for changes from baseline to follow-up for the Control group indicate that initial contact and peak knee flexion angles increased, on average by 3°, with no change for total excursion (Table 3).

**Fig. 7:**
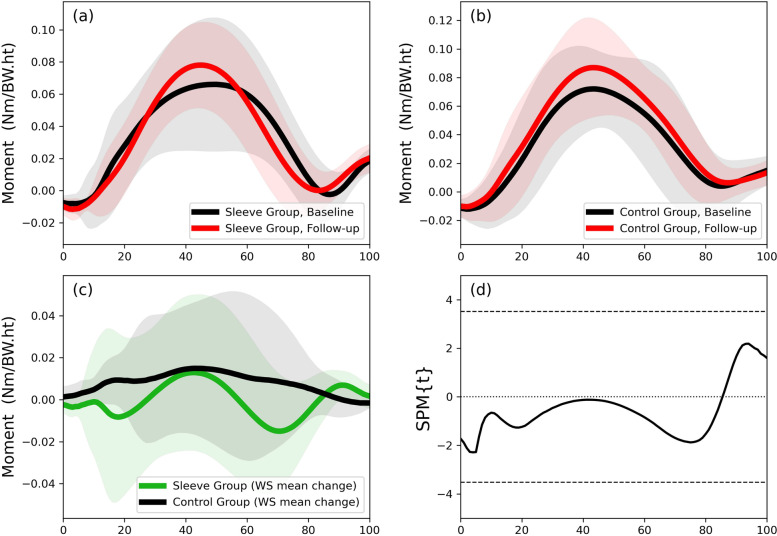
Injured side knee external flexion moment (Nm/BW.ht) trajectories during stance of the step-down-hop task for the (**a**) intervention and (**b**) control groups; (**c**) within-participant mean differences between baseline and follow-up for the intervention and the control groups; (**d**) between-group SPM analysis (independent t tests) of the baseline to follow-up mean changes. (WS: within-subject)

Knee external flexion moment trajectories for the Sleeve Group and Control Group are shown in Fig. 7 at (a) baseline and (b) six-week follow-up. The SPM analysis of the baseline to follow-up mean changes showed no between-group differences during stance phase (Fig. 7d), also reflected in lack of differences for discrete peak flexion moments (Table 3). 

**Table 3:** Randomised Clinical Trial: Parameters of injured sides at baseline and follow-up, and between-group differences of changes from baseline to follow-up

	**Control Group** **Mean (SD)**		**Sleeve Group** **Mean (SD)**		**Change score group** **Mean difference (95% CI)**		**Between group difference**
	**BL**	**FU**	**BL**	**FU**	**Control**	**Sleeve**	***p*** -value*
Flexion angle at initial contact (°)	11.1 (5.3)	14.1 (6.8)	11.8 (4.7)	13.0 (6.6)	3.0 (0.8, 5.1)	1.1 (-1.5, 3.7)	0.310
Peak flexion angle (°)	41.2 (5.9)	43.9 (6.5)	43.2 (5.6)	40.0 (8.0)	2.8 (0.1, 5.5)	-3.2 (-8.8, 2.5)	0.058
Flexion/extension Excursion (°)	30.0 (2.9)	29.8 (4.2)	31.3 (5.3)	27.0 (7.4)	-0.2 (-3.1, 2.7)	-4.3 (-11.1, 2.4)	0.627
Peak external flexion moment (Nm/BW*HT)	0.086 (0.031)	0.097 (0.027)	0.081 (0.034)	0.081 (0.026)	0.011 (-0.015, 0.036)	0.000 (-0.025, 0.025)	0.402
Stance duration (ms)	344 (56)	346 (65)	402 (89)	313 (49)	2 (-35, 38)	-89 (-153, -24)	0.013

*BL* Baseline, *FU* Follow-up; * Mann–Whitney Test

#### Temporal variable

Stance duration changed to a greater extent for the Sleeve Group than the Control Group from baseline to follow-up (Table 3). For the stance group, the average duration decreased by 22%.

## Discussion

We determined immediate and six-week effects of wearing a knee sleeve on knee kinematics and kinetics in participants with an ACL reconstruction during a sub-maximal step-down-hop task. When unsleeved, the injured side had lower external knee flexion moments and flexion angles during stance of the step-down hop compared to the uninjured side. When wearing the sleeve on the injured side, knee flexion increased to similar magnitudes as the uninjured side, particularly within the first 40% of stance. The injured side knee flexion moments remained unchanged in the immediate intervention with the knee sleeve. Thus, the first research hypothesis (H1) was accepted for increased knee flexion angles when wearing the sleeve, but not for the flexion moments based on the SPM analysis. Following the six-week period, within-participant changes from baseline to follow-up differed between the two groups: the Sleeve Group appeared to have less knee flexion at follow-up, whereas the Control group who had greater knee flexion. These differences applied to most of the stance phase. Thus, we found no support for our second research hypothesis (H2) that the Sleeve Group would have increased knee flexion angles and moments at follow-up. The analysis of stance duration suggests significantly different responses between the groups: while the Control group had no change in stance duration, the Sleeve group had a shorter stance duration at follow-up, with the time decreasing by 22% compared to the baseline duration. The Sleeve Group thus performed the task faster, possibly indicating overall enhanced performance. Thus, we found support for our third research hypothesis of decreased stance duration for the Sleeve Group at follow-up.

### Immediate responses

Our SPM analysis showed that when compared to the contralateral side, participants landed with reduced knee flexion at 35 to 65% of the stance phase when unsleeved. Peak knee flexion occurs during that period. When wearing the sleeve, the knee flexion of the injured side increased significantly during the first 40% of stance phase, corresponding to roughly the first 180 ms. ACL ruptures are likely to occur in the first 50 ms following landing [36], thus, we can speculate that enhanced flexion during that phase when wearing the sleeve may have a protective effect.

Reduced knee flexion angles during jump landing have been explored extensively in terms of risk for ACL rupture and as outcomes of such injury and reconstruction [8, 9]. Knee flexion contributes to absorption of impact forces when landing, along with hip flexion and ankle dorsiflexion [37]. Decreased angles (thus a more extended knee) are suggested to indicate ‘stiffer’ knee landing patterns, and are proposed to expose the knee joint to higher forces [8, 14]. Decreased flexion angles are proposed to be due to due to quadriceps weakness or inhibition [9, 38], or decreased ability to flex the knee during loading [39].

A clinically meaningful increase in knee flexion angles has to date not been defined [9], and may vary throughout the recovery phase. These most likely depend on the overall excursion during the specific task. Previous laboratory-based studies showed that wearing a sleeve can enhance knee flexion angles and influence frontal plane biomechanics during walking in participants with knee osteoarthritis [17, 18]. Knee flexion angles, however, increased at an average of 1.2° [17], and knee adduction angles decreased by 1.9° at initial ground contact and at 1.5° at peak [18]. Ericksen, et al. [40] reported an increase of 10° flexion when providing real-time feedback to healthy women during double-leg jump landing. However, using the same double-leg jump with a mixed group knee-healthy men and women, Welling et al. [14] found differences of around 2°, on average, with provision of various forms of verbal feedback. Those studies entailed maximal vertical jump, leading to peak knee flexion ranging between approximately 60 to 70° [14, 40]. In comparison, we used a sub-maximal horizontal hop with an average of 46° peak flexion for the uninjured side. Yet, we found a mean difference between the injured and uninjured side of 4°. Wearing the sleeve increased flexion angles at initial contact and at its peak by 3°. Participants did not focus on their knee movement patterns on landing, instead were prompted to step down and jump forwards ‘as fast as possible’. The injured side average peak flexion increased when wearing the sleeve despite their attention not being on the knee position or movement, at baseline. Thus, wearing a knee sleeve could potentially be used as an adjunct to jump-landing training. Mechanisms underlying those changes are likely due to the tactile sensation of the knee sleeve improving proprioception or awareness of the knee, leading to greater confidence with undertaking the physical task [16–18].

The SPM analysis showed that external knee flexion moments were lower for the injured side compared to the uninjured side. The discrete variable analysis showed a between-side 24% difference for peak flexion moments. Such reduced peak knee flexion moments during various types of jumps when comparing ACL-reconstructed knees with healthy controls and compared to the contralateral limb have also been reported previously [8, 9]. While the SPM showed no significant differences for the injured side flexion moment trajectory between sleeved and unsleeved, the discrete variable analysis suggests a significant increase of 7%. Differences in the results between SPM and discrete variable analyses may relate to the fact that the peak moments do not necessarily occur at the same time point during stance phase for each participant. Comparing time-based trajectories (as with the SPM) ensures magnitudes at equivalent events are compared, whereas discrete variables introduce potential covariation with event timing. Despite the small increase for the injured side flexion moment, it remained substantially lower compared to the uninjured (unsleeved) side. Furthermore, our test–retest reliability data with 10 knee-healthy participants suggests a standard error of measurement to be 6% for peak external knee flexion moments. Thus, we cannot rule out that the differences observed for knee flexion moments between the sleeved and unsleeved conditions may be due to measurement error.

### Six-week responses

The immediate responses to wearing the sleeve contrast with the six-week effects, suggesting less knee flexion for the Sleeve group, and improved knee flexion for the Control group. We tested all participants at follow-up only in the ‘no sleeve’ condition as, in clinical practice, individuals would be expected to wean themselves off use of the sleeve. Perhaps those participants had accommodated to wearing the knee sleeve daily (self-reported median 1 ½ hrs, range 42 min to 7 h [24]). Consequently, being tested at follow-up only without the sleeve may have led to an ‘unfamiliar’ condition, despite allowing time for re-familiarisation with the required task in the laboratory.

At six-week follow-up, the change scores for stance duration were different between the two experimental groups: while the Control group showed no difference in duration, the Sleeve group had shorter duration (exceeding the SDD, Appendix). The shorter stance duration at follow-up for the Sleeve Group, with less knee flexion and no changes in knee moments may indicate enhanced ability to attenuate landing forces and to generate power for the forward hop. Participants were instructed to ‘jump forward as fast as possible’, thus shorter durations indicate an improvement. Faster movement without changes in knee flexion moments (as observed for the Sleeve Group) must result in no change in flexion angle. We reported previously [24] that the Sleeve Group had higher self-reported physical activity than the Control group, but no between-group differences were found for self-reported function (IKDC-SKF), hop distance and thigh muscle strength. Increased physical activity levels reported by participants of the Sleeve Group may have improved knee function, potentially leading to enhanced power generation associated with improved movement efficiency. In combination, improved physical activity level and sensorimotor effects of the knee sleeve may have improved confidence in the knee [16–18], evident in faster action during the individual-specific standardised forward hop.

Lack of improved knee flexion angles across a few weeks has also been reported elsewhere: a 4-week plyometric training programme did not lead to enhanced movement patterns in ACL-reconstructed participants [15]. Speculative clinical implications of our findings are that use of the knee sleeve may be an adjunct to re-train jump landing patterns. While immediate increased knee angles (landing with ‘softer’ knees) when wearing the sleeve may indicate a preferred response, individuals may need to be cautioned that they also should undertake physical activity without wearing the sleeve. The knee sleeve might be a facilitator for preferred movement patterns, but to transfer those patters to daily physical activity, training without such sleeves should also be advised.

We explored the IKDC-SKF, isokinetic thigh muscle strength, the maximum horizontal single-leg hop [24] and biomechanical measures during the sub-maximal step-down hop. The stance duration of the latter was the only significant change for the Sleeve Group from baseline to follow-up. Thus, it appears that the only significant change was related to movement efficiency, in the absence of change in self-reported function and the maximum physical performance tests. Further analyses of knee power during the task and of the GRFs are warranted.

### Methodological considerations

This investigation was a secondary analysis of the influence of wearing a knee sleeve on various movement-related variables. The study was affected by the six-week COVID-19 lockdown of 2020 in New Zealand, losing 8 participants for the laboratory follow-up session. Due to time limits for this study, we were not able to recruit sufficient participants to meet the planned sample size of 16 per group. Our results may reflect a Type 2 error for the six-week effects of wearing a sleeve, thus our results may under-report actual between-group differences.

We did not explore possible compensatory movements at the trunk, hip, ankle and foot. Here we analyse movement patterns only in the sagittal plane as we found low reliability for knee kinematics and kinetics in the transvers and frontal plane during the step-down task. Our test–retest reliability data indicates a SDD of 4° for knee flexion (Appendix). The mean differences for the immediate effects and for the Control group at follow-up were 3°, thus less than the SDD. The mean difference for the stance phase for the Sleeve Group at follow-up was larger than the SDD (73 ms).

Various confounding factors need to be considered. Movement patterns differ between men and women [41] and are likely to change over the course of recovery and long-term following ACL reconstruction [8, 9]. We included participants with a large range for duration since reconstruction as knee sleeves may be used by such individuals at any stage of the rehabilitation and in the long term. The type of graft (hamstring versus patella tendon graft) may also influence the variables [42]. We thus adjusted the linear mixed models for Part 1 for sex, duration since surgery, surgery type and sequence effect. Participants were stratified by sex for the RCT. We could not adjust the analysis of Part 2 due to the small sample size.

A strength of our analysis is that our primary analysis was based on SPM to explore differences between trajectories across the entire stance phase. We report post hoc discrete variable analysis to allow comparison with other studies. A limitation of discrete variable analyses is that they only report one time-point in the entire movement. Furthermore, discrete variable analysis ultimately leads to multiple comparisons, with the risk of Type I errors. We report the *p*-values, as well as mean differences (and 95% CIs) between conditions (Part 1) and groups (Part 2) to allow the reader to interpret the outcomes.

## Conclusions

In a group of 31 participants with ACL reconstruction, knee flexion angles increased during the first 40% of the stance phase during a sub-maximal step-down hop task when wearing a knee sleeve compared to the unsleeved condition. There were no significant differences for external knee flexion moments between the two conditions. Wearing the sleeve for six weeks for a minimum of hour per day, did not lead to increased knee flexion angles or external knee flexion moments compared to the control group who did not receive that sleeve. However, stance duration decreased significantly for the Sleeve Group compared to the Control Group, potentially indicating faster or enhanced physical performance during that task. The knee sleeve might be used as an adjunct to rehabilitation to increase knee flexion angles. Prescription of such sleeve should be based on individualised assessment of immediate responses and over a defined period.

## Supplementary Information

**Additional file 1:** **Appendix 1.** Reliability of discrete variable analysis (knee-healthy participants, n=10).

## Data Availability

The datasets generated and/or analysed during the current study are not publicly available as they form part of ongoing research but are available from the corresponding author on reasonable request.

## Abbreviations

ACL: Anterior cruciate ligament
BMI: Body mass index
GRF: Ground reaction force
ICC: Intraclass correlation coefficient
IKDC-SKF: International Knee Documentation Classification Subjective Knee Form
RCT: Randomised clinical trial
SDD: Smallest detectable difference
SEM: Standard error of measurement;
SPM: Statistical parametric mapping
