# Supplementary material for: Immediate and six-week effects of wearing a knee sleeve following anterior cruciate ligament reconstruction on knee kinematics and kinetics: a cross-over laboratory and randomised clinical trial
Source: BMC Musculoskelet Disord. 2022 Jun 10;23:560. doi: 10.1186/s12891-022-05488-2 (PMC9186011; doi:10.1186/s12891-022-05488-2)
Supplement: Supplementary file 1 — Additional file 1: Appendix 1. Reliability of discrete variable analysis (knee-healthy participants, n=10). [file 12891_2022_5488_MOESM1_ESM.docx]

**Appendix 1: Reliability of discrete variable analysis (knee-healthy participants, n=10)**

|  | Day 1 Mean (SD) | Day 2 Mean (SD) | ICC (95%CI) | SEM | SDD |
| --- | --- | --- | --- | --- | --- |
| Peak knee flexion angle | 48.7° (5.0) | 49.1° (4.7) | 0.916 (0.700 – 0.978) | 1.5 | 4.0 |
| Peak external knee flexion moment (Nm/BW.ht) | 0.126 (0.025) | 0.131 (0.024) | 0.919 (0.510 – 0.951) | 0.007 (5.7%) | 0.020 (15.8%) |
| Stance duration (ms) | 350 (78) | 341 (62) | 0.888 (0.615 – 0.971) | 26 | 73 |
| ICC: Intraclass Correlation Coefficient; SD: standard deviation SDD: smallest detectable difference; SEM: standard error of measurement; 95%CI: 95% confidence interval | | | | | |
